# Unusual case of gastro jejuno-colic fistula with perforation: a rare case report

**DOI:** 10.11604/pamj.2014.18.341.5106

**Published:** 2014-08-27

**Authors:** Sundeep Ashokkumar Naik, Srinivas Pai

**Affiliations:** 1 Department of General Surgery, SDM College of Medical Sciences & Hospital, Dharwad, Karnataka, India

**Keywords:** Gastrojejunocolic fistula, gastric resection, gastrectomy, acid peptic disease

## Abstract

Gastrojejunocolic fistula (GJF) is a late and very rare complication of gastroenterostomy performed for recurrent peptic ulcer disease. The occurrence of perforation in a GJF is even more a rare complication because long evolution time or latent period is required for its appearance. Patients with this condition usually present with diarrhea, weight loss, feculent vomiting, under-nutrition and features of peritonitis that require immediate surgical intervention. Herewith we report a case of 60 year old male with perforation in a gastrojejunocolic fistula and its management.

## Introduction

Gastrojejunocolic fistula is a rare complication of surgery for peptic ulcer disease. Free perforation of a primary, benign, gastrocolic fistula is extremely rare; perforation of a gastrojejunocolic fistula has not been reported previously in the English medical literature. As per the experience with Schein M, two such cases were presented and the surgical management of this condition was emphasized [1]. Most patients with GJF present with a symptom triad of fecal vomiting/breath, chronic diarrhea, and weight loss [2, [3] The causes for a Gastrojejunocolic fistula (GJF) include - post peptic ulcer disease surgery - inadequate gastric resection or incomplete vagotomy, malignancy, tuberculosis, trauma, diverticulitis: With today's improved surgical technique and equipment, the incidence of GJF has gone down and now only occurs in approximately one out of seven individuals with marginal or recurrent ulceration [4].

## Patient and observation

A 60 year old male presented to casualty with complaints of pain abdomen and feculent/bilious vomiting over the last four days in the background of anorexia and significant weight loss over the preceding 6 months. There was history of diarrhea on and off. No malaena or haematemesis. He had undergone surgery for Acid Peptic Disease in 1994. He had no other significant co-morbidities and was on Tab. Ranitidine 150 mg BD. On examination he was conscious, co-operative and well oriented but seemed poorly nourished. He presented in a setting of hypovolaemia with a weak pulse of 90/min and a BP of 100/70 mmHg, random blood sugar was 100mg/dl. A vertical midline scar was present over the abdomen and he had mild tenderness in RIF and rt. lumbar region and other systems were normal. Preliminary investigations sent-Blood counts, renal function tests, X-Ray abdomen erect, chest X-Ray, Ultrasound of abdomen were all found to be within normal limits. He was resuscitated with IV fluids and was put on intra-venous antibiotics and Pantoprazole.

**Figure 1:**
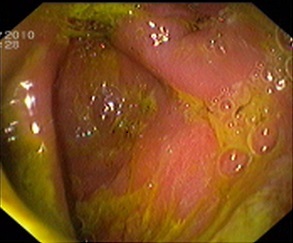
Endoscopic view of the fistulous communication between stomach and colon. Stomach filled with feculent matter

The patient started to improve over the next couple of days in the wards and a endoscopy was scheduled which showed stomach filled with fecal matter and an opening suggestive of fistulous communication to colon (Figure 1). Endoscopy also revealed gastrojejunostomy with multiple stomal ulcers. A biopsy was also taken at this point and malignancy was ruled out. After a week, he developed severe diarrhea, vomiting, appeared dehydrated and had tachycardia (104bpm) with a low volume pulse, BP was 90/70mmhg. Abdomen was diffusely tender and tense. X-Ray abdomen was repeated and it revealed gas under the diaphragm s/o pneumoperitoneum. The patient was prepared for an exploratory laparotomy.

**Figure 2:**
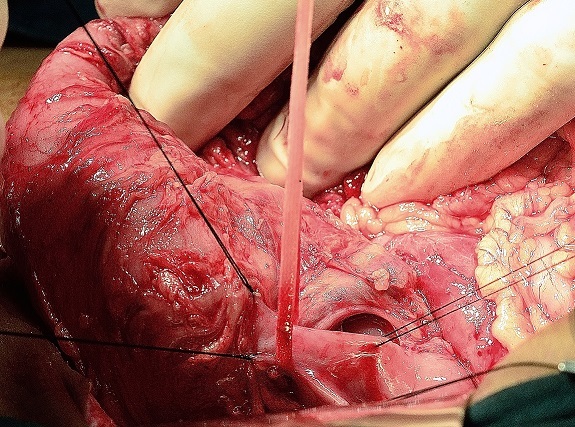
Intraoperative picture of the fistulous track between stomach, jejunum, and transverse colon

Intra-operative findings: Previous GJ stomal ulcer had given way and had extended to involve the colon (Figure 2) Peritoneal contamination in pelvis and paracolic gutters. Gastrojejunostomy was undone and transverse colon found to be involved. Fistula dissected circumferentially and wide excision of stomal ulcer and involved colon done (Triple Resection). Bowel continuity was restored with Colocolostomy, Roux-en-Y gastrojejunostomy with feeding jejunostomy. Post operative period was uneventful. Gradually the patient improved and was started on a high protein diet and was discharged on the post operative day 27. Patient is on regular follow up.

## Discussion

Gastric surgery for peptic ulcer disease is now rarely performed due to the development of medical therapies including H2-receptor antagonists, proton pump inhibitors, and regimens for *Helicobactor pylori* eradication. Gastrojejunocolic fistula is thought to be the late complication of inadequate surgery resulting from simple gastroenterostomy, inadequate gastric resection, or incomplete vagotomy [2]. The diagnosis for GJF is usually fairly simple and can be made on history and clinical examination in most cases. Investigations to aid in diagnosis today include UGI endoscopy (usually enough), Barium enema (used to be the mainstay), NG tube aspiration of faeculent material, CT with oral contrast (rarely needed), Biopsy showing colonic mucosa in the fistula is confirmatory [5].

In the above case, the initial differential diagnosis included Recurrent ulcer, Stump Carcinoma and Retrograde jejunogastric intussusception. Over the years the surgical management of gastrocolic and gastrojejunocolic fistulae has varied a great deal, ranging from - simple colostomy to three-stage procedures. It should be noted that all of these procedures have one thing in common - the diversion of the fecal stream away from the upper gastro- intestinal tract, which allows the small intestine to function normally. An important aspect of the management of these patients is total parenteral nutrition or total enteral nutrition. Due to the poor nutritional status of patients with GJF, operative mortality following surgical repair used to be as high as 40%. Staged repair of GJF, with preliminary diversion colostomy, was favored to minimize mortality [2, 3, 6, 7].

This cannot be overemphasized that, it is important to remember that in parts of the world where these therapies arrived late on the scene, gastric surgery was the mainstay of therapy even well into the 1990's. With the natural history of GJF usually requiring a few years to develop, it is not hard to imagine that these cases can appear in today's practice in the 3rd world countries especially although even in these areas these cases are quite rare. The majority of these patients will present with the classic symptoms of diarrhea, weight loss and feculent vomiting and will have some previous history of gastric surgery for peptic ulcer disease. Laboratory findings commonly reflect a state of severe malnutrition and dehydration with electrolyte imbalance, diminished serum proteins and vitamin deficiencies. A mild to moderate anemia may be present which may not be observed because of hemoconcentration [8].

Today the most common modality of treatment is a single stage Triple Resection procedure involving the entire involved area with an adequate margin followed by primary anastomosis and thorough peritoneal lavage. Patient must be put on long term PPIs or H2-antagonists post-operatively. A high recurrence rate is common if the predisposing factors for stomal ulcer are not addressed [3]. Hence the treatment must include the treatment of these risk factors.

Since the fistula formation needs a 20 to 30 years of latent period after initial surgery, this complication is seen now a days [9]. Although plenty of literatures on GJF have been published, but only few cases of perforation in GJF and its management are described. Modern management of GJF is via a one-stage resection. Masashi Takemura and all have concluded that today, laparoscopic-assisted one-stage en bloc resection may be feasible for patients with GJC fistula [10].

## Conclusion

GJF is a rare complication of gastric surgery for APD and must be kept in mind whenever a patient comes with a history of faeculent vomiting, significant weight loss and diarrhea. Diagnosis is almost always made by a UGI endoscopy and can be confirmed by histological examination of the fistula. Perforation in GJF is extremely rare condition and presents with features of peritonitis. High index of suspicion is required to diagnose perforation in GJF. The patient almost always has severe nutritional and electrolyte imbalances and thus these must be corrected along with the reparative surgery, which involves adequate resection and primary anastomosis in a single-stage procedure. In our case the final histopathology report showed chronic peptic ulcer at GJ stoma with perforation, with chronic inflammatory changes in adherent small intestine and colon, with no e/o malignancy. Off-late the incidence of GJF is becoming increasingly rare due to the advances in medical management of APD. GJF still remains a rare and interesting complication particularly in areas where gastric surgery was the primary modality of treatment few years back.

## References

[1] Schein M (1987). Free perforation of benign gastrojejunocolic and gastrocolic fistula: report of two cases. Dis Colon Rectum..

[2] Mathewson C (1941). Preliminary colostomy in the management of gastrocolic and gastrojejunocolic fistulae. Ann Surg..

[3] Lowdon AG (1953). Gastrojejunocolic fistulae. Br J Surg..

[4] Marshall SF, Knud-Hansen J (1957). Gastrojejunocolic and gastrocolic fistulas. Ann Surg..

[5] Chung (2001). Diagnosis and current management of gastrojejunocolic fistula. HKMJ.

[6] Pfeiffer DB (1941). The surgical treatment of gastrojejunocolic fistula. Surg Gynecol Obstet..

[7] Sorensen BM (1969). Non malignant gastrointestinal shortcircuit- (Gastrojejunocolic fistula and gastroileostomy). Acta Chir Scand Suppl..

[8] Puia IC, Iancu C, Bãlã O, Munteanu D, Al-Hajjar N, Cristea PG (2012). Gastrojejunocolic fistula: report of six cases and review of the literature. Chirurgia..

[9] Kil Hwan Kim, Ye Seob Jee (2013). Gastrojejuno-colic fistula after gastrojejunostomy. J Korean Surg Soc..

[10] Masashi Takemura (2011). One-stage laparoscopic-assisted resection of gastrojejunocolic fistula after gastrojejunostomy for duodenal ulcer: a case report. Journal of Medical Case Reports..

